# Towards robust causal inference in epidemiologic research: employing double cross-fit TMLE in right heart catheterization data

**DOI:** 10.1093/aje/kwae447

**Published:** 2024-12-10

**Authors:** Momenul Haque Mondol, Mohammad Ehsanul Karim

**Affiliations:** School of Population and Public Health, University of British Columbia, Vancouver, BC V6T 1Z3, Canada; Department of Statistics, University of Barishal, Barishal-8254, Bangladesh; Centre for Advancing Health Outcomes, University of British Columbia, Vancouver, BC V6Z 1Y6, Canada; School of Population and Public Health, University of British Columbia, Vancouver, BC V6T 1Z3, Canada; Centre for Advancing Health Outcomes, University of British Columbia, Vancouver, BC V6Z 1Y6, Canada

**Keywords:** causal inference, machine learning, double cross-fit, targeted maximum likelihood estimation

## Abstract

Within epidemiologic research, estimating treatment effects from observational data presents notable challenges. Targeted maximum likelihood estimation (TMLE) emerges as a robust method, addressing these challenges by accurately modeling treatment effects. This approach uniquely combines the precision of correctly specified models with the versatility of data-adaptive, flexible machine learning algorithms. Despite its effectiveness, TMLE’s integration of complex algorithms can introduce bias and undercoverage. This issue is addressed through the double cross-fit TMLE (DC-TMLE) approach, enhancing accuracy and reducing biases inherent in observational studies. However, DC-TMLE’s potential remains underexplored in epidemiologic research, primarily due to the lack of comprehensive methodologic guidance and the complexity of its computational implementation. Recognizing this gap, our article contributes a detailed, reproducible guide for implementing DC-TMLE in R, aimed specifically at epidemiologic applications. We demonstrate the utility of this method using an openly available clinical data set, underscoring its relevance and adaptability for robust epidemiologic analysis. This guide aims to facilitate broader adoption of DC-TMLE in epidemiologic studies, promoting more accurate and reliable treatment effect estimations in observational research.

## Introduction

The use of observational data in public health research and epidemiology has grown significantly,^1^^-^^7^ providing valuable insights into real-world scenarios where randomized controlled trials are unfeasible, unethical, or time-consuming.^8^ Correct model specification in causal inference methods is a common challenge using observational data, but it is crucial for less biased and accurate causal estimates.^9^ While traditional regression models may struggle to accurately specify the relationships between exposure, confounders, and outcomes, targeted maximum likelihood estimation (TMLE) emerges as a more advantageous approach.^10^ TMLE exhibits double robustness, ensuring unbiased causal estimates as long as one of the exposure or outcome models is correctly specified. However, the convergence rate of the estimator depends on the product of the convergence rates of the 2 nuisance models. To ensure valid statistical inference, we generally require this combined rate to approach *n*^–1/2^. If either of the models is misspecified, the overall convergence rate may be insufficient, leading to incorrect variance estimation by the influence function. This misestimation of variance results in invalid confidence intervals and hypothesis tests, even under TMLE.^11^ While the use of machine learning may sometimes compromise double robustness due to slower convergence rates, its ability to more accurately estimate nuisance parameters and reduce bias often justifies this trade-off, especially in high-dimensional or real-world data scenarios. Machine learning algorithms can model complex, nonlinear relationships and interactions in the data that traditional parametric models may overlook, reducing the risk of model misspecification. Ongoing research aims to preserve double robustness while addressing the slower convergence rates typical of machine learning algorithms, which often involve alternative procedures.^12^

Selecting the single right algorithm, be it a statistical model or machine learning, proves challenging in estimating the exposure and outcome nuisance models under TMLE for a given data set.^13^ Super-Learner, an ensemble machine learning technique, is recommended for the estimation of the nuisance models that improve the predictions by leveraging multiple algorithms.^14^^,^^15^ Super-Learner uses cross-validation to pick the best algorithms from a user-defined list and determines the optimal linear combination of these algorithms. It is recommended to include a diverse set of algorithms in the Super-Learner that includes the regression spline. If tree-based learners are used, then it is recommended to use sample splitting or related methods to avoid overfitting. The important interactions and nonlinear terms of the predictors are suggested to be added to the nuisance models also.^15^^,^^16^

TMLE is prone to overfitting, which causes bias and undercoverage, while the Super-Learner is paired with a tree-based complex and flexible machine learning algorithms in estimating the nuisance models.^16^ A double cross-fit version of TMLE (DC-TMLE) has been explained recently to incorporate the above recommendations that address the bias and undercoverage issues.^9^ The DC-TMLE divides the data set into 3 equal splits, where exposure and outcome models are estimated in 2 different splits, and treatment effect is estimated in the third split. This process of sample splitting and treatment effect estimation is repeated for a large number of times (usually 100 times) to estimate the average treatment effect. In a recent study, DC-TMLE was elucidated using a simulated data, and its performance was evaluated and compared with other methods.^9^ DC-TMLE was the outperformer in terms of bias and coverage in comparison with single robust methods and a non-cross-fit version of other double robust methods.^9^

Despite the enhanced features of DC-TMLE, its utilization remains notably restricted due to computational challenges and a lack of comprehensive guidance on applying DC-TMLE to real-world data in a commonly used software framework. The recent study^9^ explained DC-TMLE using R, as well as Python, through a simulation study, which was adapted and modified in another simulation study.^17^ However, a comprehensive step-by-step guide for the application of DC-TMLE to real-world data sets is still absent. Our objective is to provide a comprehensive tutorial in R illustrating the application of DC-TMLE to right heart catheterization (RHC) data to estimate the treatment effect of RHC on length of hospital stay.

## Methods

### Right heart catheterization study

A cohort of patients received care in intensive care units (ICUs) across 5 US teaching hospitals between 1989 and 1994. Among the 5735 patients in ICUs, 2184 received RHC treatment during the first 24 hours, while 3551 did not receive the treatment. The study examined the impact of RHC on the survival time, cost of care, intensity of care, and length of hospital stay (in days). The original study employed propensity score matching to assess the effect of receiving RHC within the first 24 hours of the ICU on the defined outcomes. The selection of variables for inclusion in the propensity score model was guided by the collective expertise of a panel consisting of 4 intensivists and 3 cardiologists.^10^^,^^18^^,^^19^

### Notations

In our application, receiving RHC (yes/no) in the first 24 hours of ICU care is the exposure ($A$). We will estimate the average treatment effect (ATE) of $A$ on the length of hospital stay $(Y),$ controlling the same set of confounders $(L)$ as utilized in the original study.^18^ We have applied the DC-TMLE framework to estimate the ATE, where the exposure and outcome models, ${f}_{\pi }(L)$ and ${f}_m\left(A,L\right),$ respectively, are defined as


$$ {f}_{\pi}\left(L=l\right)=E\left[A|L=l\right]\ \textrm{and}\ {f}_m\left(A=a,L=l\right)=E\left[Y|A=a,L=l\right] $$

${Y}^{A=a}$ denotes the outcome when $A$ is set to a specific value. The ATE is defined as the difference from the average length of hospital stay between patients who underwent RHC and those who were treated without RHC.


$$ ATE=E\left[{Y}^{A=1}\right]-E\left[{Y}^{A=0}\right] $$

### Step-by-step estimation of ATE using DC-TMLE

Assuming the fulfillment of causal assumptions,^20^ DC-TMLE has been applied to estimate the above-defined ATE through following steps (see the summary of DC-TMLE steps in Table 1).

**Table 1:** Double robust targeted maximum likelihood estimation (DC-TMLE) steps to estimate average treatment effect of right heart catheterization (RHC) on the length of hospital stay (in days).

**Steps**	**Describing the steps of DC-TMLE**
1	The data set is divided into 3 approximately equal splits ${O}_1,{O}_2,\textrm{and}\ {O}_3$.
2	We estimate exposure model $\widehat{f_{\pi }(l)}$ in ${O}_1,{O}_2,\textrm{and}\ {O}_3$.
3	We transform the outcome within [0, 1] range and estimate outcome model $\widehat{f_m\left(a,l\right)}$ in each of the 3 splits.
4	We predict the probability of receiving RHC ($A=1$) using the estimated exposure model$\widehat{\ {f}_{\pi }(l)}$ from step 2.
5	Lower and upper bounds are employed to constrain the predictions of exposure for each individual to avoid the unstable weights.
6	Outcomes under each exposure status are predicted using the estimated outcome model $\widehat{f_m\left(a,l\right)}$ from step 3.
7	The clever covariates $\hat{H}\left(a,l\right)$ are calculated from the predicted probability of exposure in step 4.
8	We calculate the influence parameter $\hat{\epsilon}$ for each split.
9	Predicted outcomes (from step 6) are updated using the influence parameter.
10	We transform the predicted outcomes (in step 9) back to the original scale of the outcome.
11	We estimate the local ATE by averaging the difference in predicted outcomes (updated) between exposure status for each split using the TMLE estimator. The average of 3 split-specific local ATEs is the global ATE.
12	Wald-type variance of local ATE is calculated for each split and is averaged to get the variance of global ATE.
13	We repeat steps $1\ \textrm{to}\ 12$ a number of times $(k)$ to reduce the sensitivity of particular sample splitting. The overall ATE is the median (or mean) of $k$ global ATEs. The variance of the overall ATE was the median (or mean) of within-split variance (calculated in step 12) plus the between-split variance (squared difference of overall ATE from global ATE).

#### Step 1: Divide the data set into 3 splits

The analytic data set ($Y,A,L$) is divided into 3 approximately equal splits, ${O}_1,{O}_2$, and ${O}_3$. Each patient contributes to 1 split only. First, the continuous outcome, length of hospital stay ($Y$), is transformed within the range $\left[0,1\right]$.^10^^,^^21^ A min-max normalization can transform the original outcome to ${Y}^{\ast }$. The difference between the conditional expectation of ${Y}^{\ast }$ for exposed and unexposed, given the confounders, is the ATE. The ATE is estimated on the transformed scale $AT{E}_{Y^{\ast }}$ that is later back-transformed to get the ATE in the original scale $AT{E}_Y$.


\begin{align*} AT{E}_{Y^{\ast }}&=E\left[{Y^{\ast}}^{A=1}|L=l\right]-E\left[{Y^{\ast}}^{A=0}|L=l\right]\ \textrm{and}\\ \quad AT{E}_Y&=\left[\max (Y)-\min (Y)\right]\times AT{E}_{Y^{\ast .}} \end{align*}

where, $\max (Y)$ and $\min (Y)$ are the maximum and the minimum value of original outcome $Y$. For a binary outcome, no transformation is necessary. The ATE is calculated using the following equation similarly:


$$ AT{E}_{Y_b}=E\left[{Y}_b^{A=1}|L=l\right]-E\left[{Y}_b^{A=0}|L=l\right] $$

The preparation of analytic data “ObsData” is described in the Appendix. The analytic data “ObsData” consists of 5735 patients, and each patient has exposure $A$, original outcome $Y$, and $49$ covariates ($L$). We defined “min.Y” and “max.Y” as the minimum and maximum days of the hospital stay, respectively, in the original scale. The original outcome “Y” was transformed to “Y.star,” bounded by $\left[0,1\right]$. The entire analytic data set was partitioned into 3 nearly equal subsets, with roughly 1911 patients in each split.

#### Step 2: Estimating exposure model $\widehat{{\boldsymbol{f}}_{\boldsymbol{\pi}}\!\left(\boldsymbol{L}\right)}$

The binary exposure $A$ is modeled by all potential confounders $L$. We assume strong collinearity and interaction among the variables of $L$ while modeling $A$. To capture the relationship between $A$and $L$, we have applied a combination of flexible machine learning algorithms, such as generalized linear models with least absolute shrinkage and selection operator (LASSO), random forest, and extreme gradient boosting learners under the 10-fold cross-validated Super-Learner framework.^13^^,^^14^ The LASSO model handles overfitting, whereas random forest and extreme gradient boosting capture interactions and nonlinear associations among the variables in $L$.^22^^-^^24^ We estimated the exposure models $\widehat{f_{\pi }(l)}$ in 3 splits ${O}_1,{O}_2,$ and ${O}_3$ under the above-described Super-Learner framework.

#### Step 3: Estimating outcome models $\widehat{{\boldsymbol{f}}_{\boldsymbol{m}}\!(\boldsymbol{a},\boldsymbol{l})}$

We modeled our transformed continuous outcome ${Y}^{\ast }$ by the exposure $A$ and defined confounders $L$. Likewise, in the exposure model, we utilized the same set of learners to capture the potential collinearity and nonlinear association between ${Y}^{\ast }$ and exposure-confounder set $\left(A,L\right)$. Based on the assumption of a relationship between outcome and exposure-confounder set $\left(A,L\right)$ for a specific data set, a different set of learners can be applied compared to exposure model. We applied a 10-fold cross-validated Super-Learner framework to estimate $\widehat{f_m\left(a,l\right)}$ in 3 splits. Since we have a continuous outcome, we set the “family” parameter as “gaussian” in the Super-Learner framework. For a binary outcome in other applications, the “family” parameter in the Super-Learner framework needs to be set as “binomial()” instead of “gaussian.”

#### Step 4: Predicting the probability of receiving RHC

The estimated exposure model $\widehat{f_{\pi }(l)}$ of each split has been applied to predict the exposure status of the whole data set. While the predicted exposures from discordant splits will be utilized during ATE calculation (in future step), we predicted the exposure status for the whole data set due to computational benefits. We defined 3 variables, “pi1,” “pi2,” and “pi3,” as the probabilities of receiving RHC in whole data using the 3 estimators from 3 splits, respectively. The variable “pi1” is the predicted probability where the predictor $\widehat{f_{\pi }(l)}$ was estimated in ${O}_1$. Likewise, “pi2” and “pi3” are the predicted probabilities for the whole data set, and the associated predictors $\widehat{f_{\pi }(l)}$ were estimated in ${O}_2$ and ${O}_3$ splits, respectively. These 3 variables were merged with analytic data set “ObsData.”

#### Step 5: Bounding the prediction to avoid positivity assumption

The predicted probabilities of the exposure have the potential to reach the extreme value 0 or 1 or very close. This extreme prediction violates the positivity assumption or unstable exposure weights. To avoid these circumstances, the probabilities are constrained to values close to, but not precisely equal to, 0 and 1. We have applied $5/\left[\sqrt{n}\log (n)\right]$and 1 – $5/\left[\sqrt{n}\log (n)\right]$ as the lower and upper bounds of the predictions, respectively, since they provide the optimum mean square error of the TMLE estimates and are the default bounds in the “tmle” R package.^25^^,^^26^ This bounding will mitigate the extreme prediction and instability of weight calculation.

#### Step 6: Prediction of the outcome under each exposure status

We predicted the outcomes (“mu1,” “mu2,” and “mu3”) in original data where predictors $\widehat{f_m\!\left(a,l\right)}$ were estimated in ${O}_1,{O}_2$, and ${O}_3$ splits, respectively. To predict the outcomes under both exposure status applying the estimated outcome models $\widehat{f_m\!\left(a,l\right)}$ in 3 different splits, we created 2 counterfactual populations imposing everyone exposed (“ObsData1”) and unexposed (“ObsData0”). The counterfactual data set of exposed, “ObsData1,” is used to predict their outcomes (“mu1_1,” “mu1_2,” “mu1_3”) where ${O}_1,{O}_2$, and ${O}_3$ splits were used to estimate the predictors. Likewise, “mu0_1,” “mu0_2,” and “mu0_3” are the predicted outcomes from “ObsData0.” We have restricted the predicted initial outcomes with a lower limit of 0 and an upper limit of 1 to avoid the undefined prediction (eg, default no-bounding option chosen for Q-bound).^25^ These 9 variables of predicted outcomes are merged with the analytic data set “ObsData.”

#### Step 7: Estimating the clever covariate

Clever covariates $\hat{H}\left(A=1,l\right)$ for exposed and $\hat{H}\left(A=0,l\right)$ for unexposed are calculated from the probabilities of exposure, which are very similar to the inverse probability of treatment weight.^27^^,^^28^ We created 3 clever covariates, “H1_1,” “H1_2,” and “H1_3,” by dividing the exposure status $A$ by “pi1,” “pi2,” and “pi3,” respectively. Likewise, the clever covariates “H0_1,” “H0_2,” and “H0_3” for unexposed were generated by dividing $\left(1-A\right)$ by the complement of “pi1,” “pi2,” and “pi3,” respectively (ie, the predicted probabilities of unexposed).

#### Step 8: Calculation of the influence parameter $\hat{\boldsymbol{\epsilon}}$

We calculated 2 influence parameters $\hat{\epsilon}=\left({\hat{\epsilon}}_1,{\hat{\epsilon}}_0\right)$ associated with the clever covariates $\hat{H}\left(A=1,l\right)$ and $\hat{H}\left(A=0,l\right)$ for each split. To calculate the $\hat{\epsilon}$ for any specific split (eg, ${O}_1$), we modeled the outcome variable ${Y}^{\ast }$from ${O}_1$ by the clever covariates as the independent variable and logit of the predicted outcomes as the offset. However, the predictors associated with the clever covariates and the predicted outcomes were estimated in ${O}_2$ and ${O}_3$ splits, respectively. Alternatively, instead of adding the clever covariates as independent variables, one could use them as weights in the model. This method is asymptotically equivalent to the earlier approach but is thought to perform better with finite samples.^21^^,^^29^ Likewise, ${O}_3$ and ${O}_1$ splits were utilized to estimate the prediction algorithms while modeling ${Y}^{\ast }$ in ${O}_2$. Again, we utilized discordant splits ${O}_1$ and ${O}_2$ to estimate the prediction algorithms while modeling ${Y}^{\ast }$ in ${O}_3$. The estimated influence parameters are denoted as $\epsilon 1=\left(\epsilon{1}_1,\epsilon{1}_0\right),\epsilon 2=\left(\epsilon{2}_1,\epsilon{2}_0\right),$ and $\epsilon 3=\left(\epsilon{3}_1,\epsilon{3}_0\right),$ for ${O}_1$, ${O}_2,$ and ${O}_3$ splits, respectively.

#### Step 9: Updating the predicted outcome

We updated the predicted outcomes that were calculated in step 6 by utilizing the estimated influence parameters $\hat{\epsilon}$ in step 8. To update the predicted outcomes of ${O}_1$, splits ${O}_2$ and ${O}_3$ were utilized to estimate the prediction algorithms of exposure and influence parameters, respectively. Likewise, discordant splits were utilized to update the predicted outcome for the other 2 splits. We defined updated outcomes “mu1_1_Ystar,” “mu1_2_Ystar,” and “mu1_3_Ystar” for an exposed counterfactual population of ${O}_1$, ${O}_2,$ and ${O}_3$ splits, respectively. Likewise, “mu0_1_Ystar,” “mu0_2_Ystar,” and “mu0_3_Ystar” are the predicted outcomes of an unexposed counterfactual population. These 6 variables are merged with an analytic data set.

#### Step 10: Transforming the updated predicted outcome to the original scale

We transformed the split-specific predicted outcomes calculated in step 9 to the original scale. The predicted outcome for split ${O}_1$ “mu1_1_Ystar” is transferred to original scale “mu1_1_Y” by multiplying the range of $Y,\max \left(\mathrm{Y}\right)-\min \left(\mathrm{Y}\right)$, and then adding the “min(Y).” We calculated counterfactual outcomes “mu1_1_Y,” “mu1_2_Y,” and “mu1_3_Y” for exposed and “mu0_1_Y,” “mu0_2_Y,” and “mu0_3_Y” for unexposed (in original scale) for ${O}_1,$  ${O}_2,$ and ${O}_3$ splits, respectively, that were used to calculate the ATE in step 11 and its variance in step 12.^21^^,^^25^ These 6 variables were merged with our analytic data set. However, step 10 is not required for a binary outcome.

#### Step 11: Estimating the ATE

We calculated the difference in predicted outcomes from exposed to unexposed for a specific split and averaged them to estimate the split-specific or local ATE. The average of the 3 split-specific ATEs were calculated to find the global ATE for the full data set. For example, if $\widehat{\mu_s^{A=1}}$ are the split-specific updated predicted outcomes for exposed, and $\widehat{\mu_s^{A=0}}$ are for the unexposed (ie, “mu1_1_Y” and “mu0_1_Y,” respectively for $s=1$), the split-specific $AT{E}_s$ and global $ATE$ can be calculated using the following formula:


$$ AT{E}_s=\frac{1}{n}\sum_{i=1}^n\left(\widehat{{\mu_s^{A=1}}_i}-\widehat{{\mu_s^{A=0}}_i}\right);s=1,2,3;\ \textrm{and}\ AT{E}_Y=\frac{1}{3}\sum_{s=1}^3 AT{E}_s $$

#### Step 12: Estimating the variance of ATE

We calculated the variance of split-specific or local ATE from the efficient influence function.^10^^,^^19^ The efficient influence function, which can be denoted as $EIC$, is estimated based on the predicted outcomes, exposure, and ATEs using the following formula:


\begin{align*} {\sigma}_{AT{E}_s}^2&=\frac{Var\left( EI{C}_{AT{E}_s}\right)}{n}\ \textrm{and}\ EI{C}_{AT{E}_s}\\ &=\left(\frac{A}{f_{\pi}^{A=1}(L)}-\frac{1-A}{f_{\pi}^{A=0}(L)}\right)\ \left(Y-{\mu}_s^A\right)+{\mu}_s^{A=1}-{\mu}_s^{A=0}- AT{E}_s \end{align*}

The variances of local ATEs from 3 splits were then averaged to get the variance of global ATE.


$$ {\sigma}_{AT E}^2=\frac{1}{3}\sum_{s=1}^3{\sigma}_{AT{E}_s}^2 $$

We followed 4 steps to calculate the variance of global ATE: we (i) estimated the efficient influence function “if1_1,” “if1_2,” and “if1_3” for exposed in ${O}_1,$  ${O}_2,$ and ${O}_3$ splits, respectively; (ii) estimated the split-specific efficient influence function “if0_1,” “if0_2,” and “if0_3” for unexposed; (iii) estimated the difference in efficient influence function “ifd_1,” “ifd_2,” and “ifd_3” between exposed and unexposed; and (iv) calculated variance of “ifd_1,” “ifd_2,” and “ifd_3,” the variances of local ATEs, which were averaged to get the variance of global ATE.

#### Step 13: Repeat the steps 1 to 12 for $\boldsymbol{k}$ times

The steps 1 to 12 were repeated for $k=100$ times to reduce the sensitivity of any individual to a specific split.^30^ While mean can be used to summarize the 100 repetitions, we considered median due to its robustness to extreme estimates.^30^ Therefore, the median of 100 global ATEs was the overall ATE of RHC on the length of hospital stay. The variance of the overall ATE was the median of within-split variance (calculated in step 12) plus the between-split variance (squared difference of the overall ATE from the global ATE). A loop was applied to repeat steps 1 to 12 a hundred times and calculate the overall ATE and its variance. The step-specific R codes and associated results can be found in Appendix S1.

### Implementing DC-TMLE by the R package

We created an R package “Crossfit” hosted on GitHub for implementing the DC-TMLE to any real-world data set.^31^ The “Crossfit” package estimates the marginal mean difference (or risk difference for binary outcome) of a continuous outcome. The “Crossfit” package receives any continuous or binary outcomes as input and calculates the ATE, its standard error, and 95% confidence interval of the ATE. The package transforms the continuous outcome within the [0, 1] range automatically and returns the estimated ATE in the original scale. We calculated the ATE of RHC on the length of hospital stay using the “Crossfit” package. Within the package, we have provided the same set of covariates for exposure and outcome models (“covarsT” and “covarsO,” respectively). The number of splits (“n_split”) was 3, and the number of repetitions (“num_cf”) from steps 1 to 12 was set as 100 for overall ATE calculations. The “Crossfit” allows applying a diverse set of learners from the “Super-Learner” package.^32^ To apply different statistical and machine learning algorithms, such as applying random forest and extreme gradient boosting, within the “Crossfit” package, we have to install the associated R packages “randomForest” and “xgboost,” respectively. The estimated ATE of RHC on the length of hospital stays was 3.18 with a 95% confidence interval of 2.28 to 4.08 (see Appendix S2).

### Simulation results

We conducted the same simulation study as previously described^9^ to evaluate the performance of DC-TMLE, implemented through our “Crossfit” package in R. The study included 500 Monte Carlo data sets, each containing 3,000 observations. We estimated the average treatment effect using both conventional TMLE and DC-TMLE, under conditions where the nuisance models were incorrectly specified. The Super-Learner used in these models incorporated a diverse set of candidate learners: mean model, linear model, generalized additive models with splines (*df* = 4 and 6), LASSO, a neural network with 2 units in the hidden layer, and random forest with 500 trees and at least 20 individuals per leaf (a method not belonging to the Donsker class). Compared to conventional TMLE, DC-TMLE provided improved estimates of bias, model standard error, and coverage of the 95% confidence intervals (see Figures S1, S2, and S3 in Appendix S3).

### Impacts of learners on computing time and ATE estimates

We estimated the ATE of RHC on the length of hospital stays and its confidence interval utilizing the “Crossfit” package in a high-performance computing platform. The computing platform featured a single node with 50 tasks (equivalent to 50 CPUs) and 50 GB of memory. The analysis took approximately 578.6 minutes to complete, during which parallel computation was applied across the repetitions (step 13).^33^ The computing time required more than 30 hours to conduct the same analysis without parallelization. We calculated the ATE, corresponding confidence interval, and computing time under a different set of parameters, such as sample size, number of repetitions, number of folds in the cross-validation of Super-Learner, and choosing a different set of learners (see Table 2). We took 2 random subsets of 1000 and 3000 patients from the original sample of 5735 patients to explore the impact of sample size on the computing time. We required an increased amount of computing time for a larger cohort of patients for a specific set of learners. The additional learner was associated with more computing time. We applied DC-TMLE using 10 and 50 repetitions to the original cohort of patients and compared with 100 commonly used repetitions. The computing time was positively associated with the number of repetitions and additional learners. Given a specific set of learners, the computing time was positively associated with the number of folds in the cross-validation under the Super-Learner framework. Compared to serial computing (without parallelization), parallel computing reduced the computing time significantly in all cases.

**Table 2:** The required computing time (in minutes) to estimate the average treatment effect (ATE) of right heart catheterization on the length of hospital stays and associated variance under a different set of parameters. We conducted the analysis utilizing the “Crossfit” package on a high-performance computing platform, featuring a single node with 50 tasks (equivalent to 50 CPU core) and 50 GB of memory. The estimates of ATE with 95% confidence intervals (CIs), calculated computing time, applying parallelization over the number of repetitions (mentioned in parentheses), and without any parallelization are summarized here.

			**Learners utilized for exposure and outcome models**					
**n** ^**a**^	**k** ^**b**^	**v** ^**c**^	**glmnet**		**glmnet and RF**		**glmnet, RF, and xgboost**	
			**ATE** **(95% CI)**	**Comp.** **time**^**d**^	**ATE** **(95% CI)**	**Comp.** **time**	**ATE** **(95% CI)**	**Comp.** **time**
1000	100	10	3.5 (–0.1 to 7.20)	26.3 (6.12)	4.94 (2.54 to 7.34)	164.4 (44.4)	4.72 (2.30 to 7.13)	248.7 (79.8)
3000	100	10	3.8 (1.50 to 6.10)	46.9 (9.96)	4.04 (2.63 to 5.46)	784.1 (180.4)	4.00 (2.63 to 5.37)	926.7 (226.1)
5735	100	10	3.30 (1.68 to 4.92)	59.7 (14.8)	3.27 (2.32 to 4.21)	1655.5 (435.5)	3.18 (2.28 to 4.08)	1842.9 (578.6)
5735	10	10	3.37 (1.76 to 4.98)	5.61 (3.41)	3.27 (2.32 to 4.23)	207.2 (57.2)	3.22 (2.30 to 4.14)	246.9 (64.9)
5735	50	10	3.34 (1.73 to 4.96)	30.2 (9.34)	3.27 (2.32 to 4.22)	1073.7 (245.9)	3.2 (2.29 to 4.10)	1234.5 (274.3)
5735	100	5	3.31 (1.70 to 4.92)	28.3 (9.38)	3.31 (1.17 to 4.92)	1061.6 (257.5)	3.17 (2.26 to 4.08)	1538.7 (309.9)

Abbreviations: glmnet, generalized linear models with least absolute shrinkage and selection operator (LASSO); RF, random forest; xgboost, Extreme Gradient Boosting algorithm.

a Sample size of the full sample (5735 patients) and 2 random subsets (1000 and 3000 patients) taken from the full sample using the 2575 seed.

b DC-TMLE steps 1 to 12 are repeated for *k* number of times.

c Number of folds in the cross-validation procedure under Super-Learner.

d Calculated computing time.

We observed substantial variations in the estimates of ATE and its confidence interval based on the choice of learners while keeping other parameters constant. Notably, the impact of learner selection on treatment effect estimates was more pronounced in smaller patient cohorts compared to larger ones. When using a specific set of learners, we noted minimal effects on ATE estimates with changes in repetitions and the number of cross-validation folds in the Super-Learner. Nevertheless, the influence of repetitions and cross-validation folds varied among different sets of learners.

## Discussion

TMLE is an increasingly used estimator to estimate treatment effect from observational studies using available software.^14^^,^^25^ Applying statistical and machine learning algorithms to estimate the exposure and outcome models of TMLE has made it more appealing to reduce the chance of model misspecification compared to the propensity score or g-computation methods. However, TMLE may provide bias and undercoverage with the complex and flexible machine learning algorithms. DC-TMLE, integrated with a set of diverse machine learning algorithms, mitigates the so-called own observation bias and undercoverage issues of TMLE by dividing the sample into 3 splits and provides improved estimates of the treatment effect. In the intermediate steps of DC-TMLE, exposure and outcome models are estimated in 2 different splits, which are then utilized to estimate the ATE in the third discordant split. Every split is utilized to estimate exposure, outcome models, and ATE calculation once, and the whole process is repeated a large number of times to reduce the sensitivity of the specific split. While this sample splitting approach showed improved estimates in terms of bias and coverage, the application of this DC-TMLE to real-world data is challenging for practical researchers.^9^

We have applied DC-TMLE for analyzing real-world study data^18^ aiming to estimate the ATE of RHC use within 24 hours among ICU patients on their length of hospital stays. Our description of the DC-TMLE application follows a step-by-step process, providing reproducible R codes intended to guide practitioners in applying DC-TMLE to similar studies for treatment effect estimation. Additionally, we developed an R package named “Crossfit” specifically designed for applying DC-TMLE to real-world observational studies to estimate treatment effect.^31^ While software codes are available in the literature to apply DC-TMLE, the codes are less useful for a user-defined real-world data set and continuous outcome.^9^ Our software package allows both binary and continuous outcomes for any user-defined real-world data set. The crucial part of the application of DC-TMLE is the computational challenges associated with it. The current version of software codes is allowed to apply parallel computing in a high-performance computing setup, which may reduce the calculation time. Researchers can easily integrate a diverse set of statistical and machine learning algorithms under the Super-Learner framework to improve the treatment effect estimates.

DC-TMLE relies on a similar assumption of no unmeasured confounding as TMLE. Necessary variables are often unavailable in observational studies, and consequently, DC-TMLE may face model misspecification. This unmeasured confounding may provide inconsistent treatment effect estimates. Besides, the estimation of treatment effect using DC-TMLE is computationally challenging and requires a high-performance computing platform. However, parallel computing in a high-performance setup can reduce the computing time significantly. While it is suggested to include complex and flexible machine learning algorithms in DC-TMLE for improved estimates,^13^ an increased amount of time is required for a complex set of candidate learners. The current version of the “Crossfit” package requires additional efforts for parallel computing that could be challenging for practitioners. Future versions of the software can be developed, incorporating the in-built nature of parallel computing.

## Supplementary Material

Web_Material_kwae447

## Data Availability

The data set utilized for this tutorial on right heart catheterization is publicly accessible and can be retrieved, along with its documentation, from https://hbiostat.org/data/. The analysis conducted on secondary and deidentified data is exempt from research ethics approval requirements, adhering to both the University of British Columbia Policy 89 and the Tri-Council Policy Statement: Ethical Conduct for Research Involving Humans, Article 2.5. The corresponding software codes and stepwise outputs are available in the Appendix.
